# Prototypical antipsychotic drugs protect hippocampal neuronal cultures against cell death induced by growth medium deprivation

**DOI:** 10.1186/1471-2202-7-28

**Published:** 2006-03-30

**Authors:** Stéphane Bastianetto, Marc Danik, Françoise Mennicken, Sylvain Williams, Rémi Quirion

**Affiliations:** 1 Douglas Hospital Research Centre, Department of Psychiatry, McGill University, 6875 LaSalle Boulevard, Montreal, Québec, H4H 1R3, Canada

## Abstract

**Background:**

Several clinical studies suggested that antipsychotic-based medications could ameliorate cognitive functions impaired in certain schizophrenic patients. Accordingly, we investigated the effects of various dopaminergic receptor antagonists – including atypical antipsychotics that are prescribed for the treatment of schizophrenia – in a model of toxicity using cultured hippocampal neurons, the hippocampus being a region of particular relevance to cognition.

**Results:**

Hippocampal cell death induced by deprivation of growth medium constituents was strongly blocked by drugs including antipsychotics (10^-10^-10^-6 ^M) that display nM affinities for D_2 _and/or D_4 _receptors (clozapine, haloperidol, (±)-sulpiride, domperidone, clozapine, risperidone, chlorpromazine, (+)-butaclamol and L-741,742). These effects were shared by some caspases inhibitors and were not accompanied by inhibition of reactive oxygen species. In contrast, (-)-raclopride and remoxipride, two drugs that preferentially bind D_2 _over D_4 _receptors were ineffective, as well as the selective D_3 _receptor antagonist U 99194. Interestingly, (-)-raclopride (10^-6 ^M) was able to block the neuroprotective effect of the atypical antipsychotic clozapine (10^-6 ^M).

**Conclusion:**

Taken together, these data suggest that D2-like receptors, particularly the D_4 _subtype, mediate the neuroprotective effects of antipsychotic drugs possibly through a ROS-independent, caspase-dependent mechanism.

## Background

There is clinical evidence of cognitive dysfunction in certain schizophrenic patients that is likely to be independent of psychotic symptoms [1]. This dysfunction does not seem to involve a single brain region but rather a network that includes cortical and sub-cortical regions such as the hippocampus. The therapeutic benefits of various antipsychotic drugs are thought to be predominantly associated with their antagonistic actions on D2-like (D_2_, D_3 _and D_4_) dopamine receptors in the brain [2,3]. Although early studies with typical antipsychotic drugs (e.g. haloperidol, chlorpromazine) mostly failed to report significant improvements of cognitive behaviors in schizophrenic patients [4-6], more recent data especially obtained using atypical antipsychotics (e.g. clozapine, risperidone, olanzapine) demonstrated positive effects [7-12]. For example, risperidone has been associated with improved verbal working memory and executive functions whereas clozapine and quetiapine seem to improve verbal fluency [9,13,14].

The beneficial effects of antipsychotics on cognitive functions and neuroprotection are supported by *in vitro *and animal studies reporting on the protective effects of these drugs in various models of toxicity including focal ischemia [15-19], serum deprivation [20], oxidative stress [21] and apoptosis [22]. More recently, it has been reported that the antipsychotic olanzapine was neuroprotective against various forms of toxicity through the phosphorylation of kinases such as Akt [23].

In the present study, the possible neuroprotective properties of low concentrations of various antipsychotic drugs and other dopamine receptor antagonists were studied in a model of toxicity using primary cultured neurons of the hippocampus, an area particularly relevant to cognitive processes.

## Results

### Dopamine receptor transcripts are expressed in mature cultured hippocampal neurons

We estimated first the number of mature neurons in our 3-day old hippocampal cultures using immunocytochemistry for the neuron-specific marker NeuN [24]. Approximately 75% of the cells were labeled thereby indicating that a high proportion of neurons were mature at this stage.

**Figure 1:**
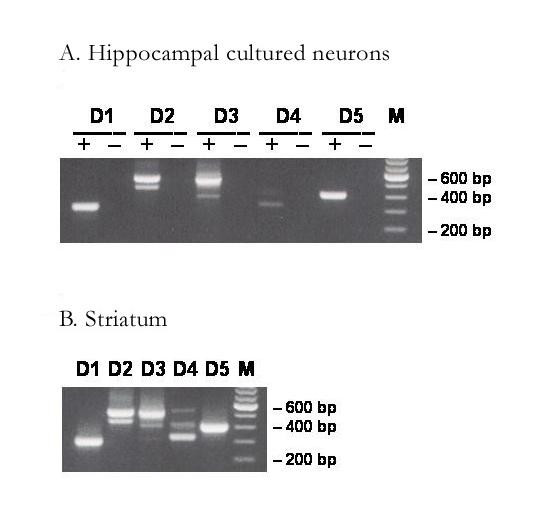
**Agarose gel electrophoresis showing RT-PCR products of dopamine receptor subtypes (D1 to D5) mRNAs in rat hippocampal neurons (A) and whole striatum (B)**. Lanes (+) and (-) represent the PCR products amplified from hippocampal neurons cDNAs following reverse transcription in the presence or absence of reverse transcriptase, respectively. The two hippocampal cultures gave identical results. Expected size for PCR products: D1, 300 bp; D2, 538 bp and 451 bp; D3, 523 bp and 410 bp; D4, 324 bp; D5, 403 bp. Lane M, molecular size standard 100-bp ladder.

We determined next if the genes coding for the dopamine receptor subtypes were expressed in these cultures. The primer pairs for the amplification of dopamine receptor subtypes 1 to 5 cDNAs were first tested on RNA extracted from rat striatum using a reverse transcription-multiplex PCR (RT-mPCR). As shown in Fig 1B, all primer pairs were able to generate products of the expected length. RT-mPCR was next performed on samples from untreated 3 day-old primary hippocampal cultures. Transcripts for all five dopamine receptor subtypes were also found to be expressed in these cultures (Fig. 1A). It is of note that band intensities do not necessarily reflect relative expression levels of transcripts for the various dopamine receptor subtypes in the starting extract since no internal standards were used. No products were seen when reverse transcriptase was omitted in the RT step indicating that amplified fragments are from transcribed mRNA. Splice isoforms for the D_2 _and D_3 _receptor subtypes were observed as well, in both striatum and hippocampal cultures. Sequencing of hippocampal main PCR products confirmed that amplifications were specific for dopamine receptors and that the D_2 _primer pair amplified the two alternatively spliced transcripts coding for functionally distinct isoforms D_2_L and D_2_S [25,26].

### Effects of typical and atypical antipsychotics against toxicity induced by N2 constituents-deprivation

**Figure 2:**
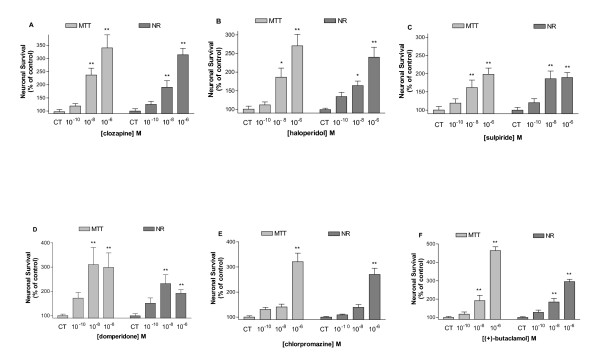
**Effects of clozapine (A), haloperidol (B), (±)-sulpiride (C), domperidone (D), chlorpromazine (E) and (+)-butaclamol (F) against toxicity induced by growth medium deprivation in enriched hippocampal neuronal cultures**. Neuronal survival is estimated using the MTT and neutral red (NR) colorimetric assays. Values represent mean ± SEM of at least three separate experiments, each performed in quadruplicate. *p < 0.05, **p < 0.01 compared to vehicle-treated groups.

As previously described in rat neuroblastoma cells [27], deprivation of transferrin, one of the major iron transport protein in the blood [28], selenium, an essential nutrient with antioxidant properties [29], as well as putrescine, a drug with growth-stimulatory properties [27], resulted in about 70 % of hippocampal neuronal cell death as monitored 3 days later using MTT and NR colorimetric assays. Cell death was strongly reduced, in a concentration-dependent manner, in presence of atypical antipsychotics such as clozapine which preferentially binds to D_4 _receptors over D_2 _or D_3 _receptors (Fig 2A) and risperidone, a D_2_/D_4 _receptor antagonist that protected hippocampal neurons at the highest concentration tested here [100 ± 6 (CT) vs 162 ± 12 (CT + risperidone 10^-6 ^M); p < 0.01]. The effects of these atypical antipsychotics were shared by the classical antipsychotic haloperidol which offered a maximal protection at 10^-6 ^M (Fig. 2B) while, as expected, a higher concentration (10^-4 ^M) was toxic on its own to hippocampal neurons (10 ± 2 vs 100 ± 4; p < 0.01). Similar effects were obtained with (±)-sulpiride, a selective D_2 _dopamine receptor antagonist belonging to the benzamide class (Fig 2C); domperidone, a D_2_/D_3 _receptor antagonist (Fig 2D); chlorpromazine, a typical antipsychotic which binds with nM affinities to D_2_, D_3_, and D_4 _receptors (Fig 2E); and (+)-butaclamol, a D_2_/D_4 _dopamine receptor antagonist (Fig 2F). Interestingly, a D_4 _receptor antagonist, L-741,742 (10^-6^M) [30] somewhat protected neurons [100 ± 5 (CT) vs 186 ± 10 (CT + L-741,742) and vs 156 ± 15 (CT + haloperidol 10^-6 ^M); p < 0.01]. Cells treated with the N2 supplement showed the same magnitude of protection (with MTT values ranging from 205% to 389% vs control groups) as that of cells treated with most of drugs at 10^-6 ^M, suggesting that depletion in growth medium rather than cell washes are responsible for decreases in MTT and NR values.

**Figure 3:**
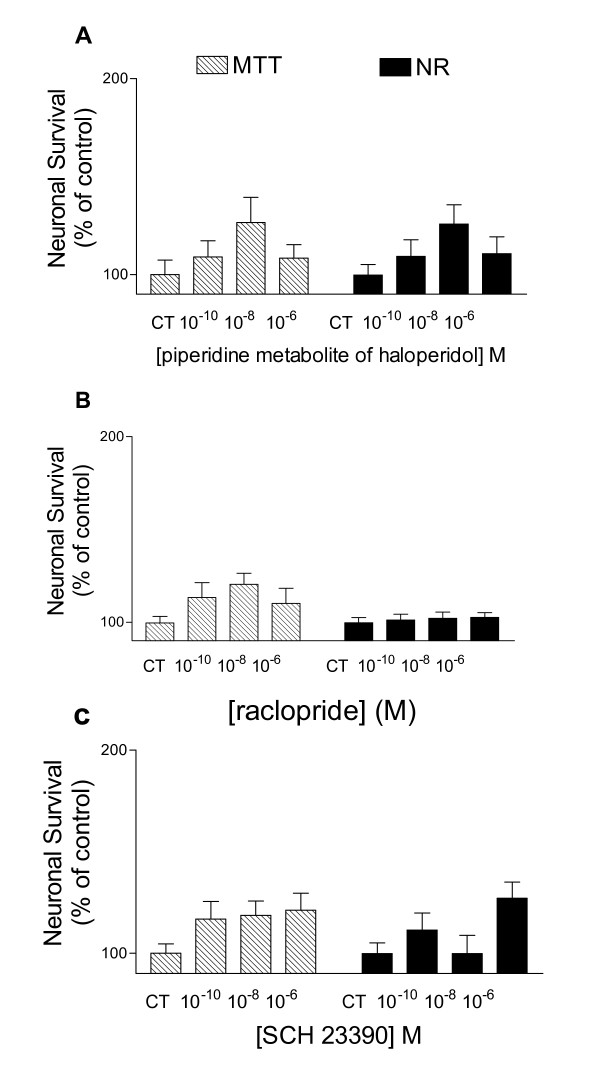
**Effects of piperidine metabolite of haloperidol (A), raclopride (B) and (+)-SCH 23390 (C) against toxicity induced by growth medium deprivation in enriched hippocampal neuronal cultures**. Neuronal survival is estimated using the MTT and neutral red (NR) colorimetric assays. Values represent mean ± SEM of at least three separate experiments, each performed in quadruplicate.

In contrast, the piperidine metabolite of haloperidol, which is devoid of affinity for D_2_-like receptor [31] was ineffective (Fig 3A). Similarly, (-)-raclopride, a D_2_/D_3 _receptor antagonist, and the D_1 _receptor antagonist (+)-SCH-23390 failed to protect hippocampal neurons (Fig 3B,C). The D_3 _dopamine receptor antagonist U 99194 maleate (10^-6^M)[32] was also ineffective [100 ± 5 (CT) vs 100 ± 6 (CT + U 99194)] (Table 1). Finally, remoxipride, another D_2 _receptor antagonist with weak D_4 _receptor affinity, failed to protect neuronal cells [100 ± 3 (CT) vs 108 ± 3 (CT + remoxipride) vs 141 ± 4 (CT + N2)].

Table 1 summarizes the apparent affinities of various dopamine receptor antagonists for the D_2 _and D_4 _subtypes with their protective effects on hippocampal neurons.

### D2 but neither sigma nor NMDA receptor antagonists blocked the protective effect of antipsychotics

Besides its well-known antidopaminergic activity, it has been hypothesized that haloperidol protects neuronal cells[16] through its purported activity at σ_1 _[33] or NMDA receptors [34]. However, neither NE-100 (10^-7^-10^-5 ^M), a potent and selective σ_1 _receptor subtype antagonist, nor (+)-MK-801 (10^-6^-10^-5 ^M), a non-competitive NMDA antagonist, affected neuronal survival (Table 2). Moreover, these compounds failed to modulate the protective effect of haloperidol (data not shown). Interestingly, the protective effect of clozapine (10^-6 ^M) was blocked by a 5-min pre-treatment with the (-)-raclopride, the sole D2-like receptor antagonist that failed to protect cells in our model (Table 2).

### The protective effects of antipsychotic drugs may involve caspases but not the inhibition of the production if free radicals

In light of the purported anti-apoptotic effects of atypical antipsychotics drugs [22], we investigated next the effects of various inhibitors of caspases, these enzymes likely playing a pivotal role in apoptosis-related cell death. In our model, the co-administration of the caspase-3 inhibitor DEVDO-CHO (5 μM), the caspase-8 inhibitor IETD-CHO (5 μM) or to a lesser extent the caspase-9 inhibitor LEHD-CHO (5 μM) significantly reduced cell death, DEVDO-CHO being the most potent (Table 3). The protective effects of inhibitors of caspases 3, 8 and 9 were not accompanied by changes in ROS accumulation, as evaluated by the DCF assay (Table 3). It has recently been shown that the atypical antipsychotic olanzapine increased cell viability after an exposure to H_2_O_2 _[21] suggesting that blockade of peroxide accumulation may be involved in the protective effects of antipsychotics reported here. However, results obtained using the DCF assay indicated that haloperidol (10^-6 ^M) did not affect intracellular ROS (in particular peroxide) accumulation whereas the well-known Ginkgo biloba extract EGb 761 that displayed potent antioxidant properties [35] strongly reduced ROS production [100 ± 3 (CT) vs 93 ± 3 (CT + haloperidol 10^-6^M) and vs 62 ± 3 (CT + EGb 761 50 μg/ml), p < 0.01]. Moreover, haloperidol and other antipsychotic drugs including (±)-sulpiride and chlorpromazine did not protect hippocampal neurons from toxicity induced by H_2_O_2 _(100 μM) in our model (data not shown).

## Discussion

Our data indicate that low concentrations of various antipsychotic drugs protect hippocampal neurons against toxicity induced by growth medium deprivation. To our knowledge, this is the first study that reports (with the exception of haloperidol) on the neuroprotective effects of various neuroleptics having high affinity for the dopamine D_2 _and D_4 _receptor subtypes in hippocampal cultured neurons. These effects are apparently not linked to the inhibition of free radical production and may involve a caspase-associated mechanism.

The protective effects of antipsychotics are not likely to be related to their inhibitory action on σ_1_- or NMDA receptor-mediated responses [33,36] since neither NE-100 nor (+)-MK-801 offered protection by themselves nor blocked the neuroprotective effects of haloperidol. On the other hand, our data suggest that D_2 _and/or D_4 _receptors mediate the effects of antipsychotic drugs in our model. First, RT-PCR data showed that D_2 _and D_4 _receptors are expressed in hippocampal neurons. These data are in agreement with previous studies reporting on the presence of these receptors subtypes in the hippocampal formation [37,38]. Second, all antipsychotics tested here (with the exception of (-)-raclopride and remoxipride) that display nM affinities for D_2 _and D_4 _receptors [40-46] were neuroprotective to hippocampal neurons. Third, (-)-raclopride, a preferential D_2 _antagonist, almost completely blocked the neuroprotective effects of clozapine, an atypical antipsychotic with a particularly high affinity for the D_4 _subtype.

A preferential role for the D_4 _receptor in the neuroprotective effect of the various antipsychotics tested in our model is of special interest. Haloperidol, risperidone, chlorpromazine, (+)-butaclamol, domperidone and clozapine exhibit high nM affinities for this receptor sub-type [39,42,43,46] and are potent neuroprotective agents in our model. Moreover, L-741,742, a rather selective D_4 _antagonist [30] was found to be neuroprotective in our model while (-)-raclopride and remoxipride which bind with only modest affinities to the D_4 _subtype [39,44] were not effective. U 99194, a potent and selective D_3 _receptor antagonist, and SCH 23390, a D_1 _antagonist, failed to be neuroprotective, suggesting that these two receptor subtypes do not mediate the protective effects of antipsychotic drugs in our model (see Table 1 for details). Interestingly, in the mature mammalian brain, the level of D_4 _receptors is greater than that of the D_2 _subtype in the hippocampal formation [37]. It would now be of interest to explore further the respective role of the D_2 _and D_4 _receptors in the neuroprotective effects of antipsychotics in hippocampal neurons using molecular approaches such as knock-out animals and siRNA. We cannot exclude however the possibility that their neuroprotective ability may also be due to their purported α_1_-adrenoceptor antagonist activity [47] which has been suggested to contribute to their clinical effect [48]. It has recently been shown that the atypical antipsychotic olanzapine attenuated cell death produced by H_2_O_2 _in PC12 cells through a mechanism that involves the upregulation of the antioxidant enzyme superoxide dismutase [21]. Although the effects of D_2_-like receptor antagonists were shared by antioxidants such as Trolox [49] and EGb 761 (data not shown), we found that they were ineffective against toxicity induced by H_2_O_2 _(haloperidol, (±)-sulpiride and chlorpromazine) and did not attenuate intracellular ROS production (haloperidol), suggesting that the protective effects of antipsychotic drugs are not due to an antioxidant activity in our model. Moreover, studies from animal models reported that olanzapine and risperidone, but not haloperidol, stimulated neurogenesis in rat brain areas (e.g. hippocampus) [50] and preserved cholinergic pathways and cognitive function, possibly by increasing levels of nerve growth factor (NGF) [51]. This suggests that the promoting effects of antipsychotics -particularly atypical ones- on neuronal function may be also due to other mechanisms including stimulatory effect on neurotrophic factors. In support of this hypothesis, it has been demonstrated that olanzapine and other atypical antipsychotics including clozapine, quetiapine and risperidone exerted protective effects in PC12 cells, possibly by decreasing the expression of the gene encoding for the neurotrophin receptor p75 [20,52]. This is of particular interest here since the p75 receptor has been reported to mediate hippocampal neuronal loss, possibly via the activation of caspases [53]. In support of an anti-apoptotic effect of antipsychotic drugs in our model, inhibitors of caspases 3, 8 and 9 were found to exert neuroprotective effects without affecting ROS production. Interestingly, it has recently been shown that clozapine and risperidone prevented apoptosis and DNA damage induced by the apoptotic agent N-methyl-4-phenylpyridinium in PC12 cells, possibly by attenuating the activation of an enzyme known as glycosylase [22].

Although only obtained using an *in vitro *model, our data are in accordance with the view that treatment with atypical antipsychotics may improve cognitive functions in schizophrenia [7-11,13,54,55]. Interestingly, only low concentrations of the various antipsychotics tested here, (with the exception of chlorpromazine that is only effective at 1 μM), were needed in our model to offer neuroprotection, much lower than those (i.e. 10–50 μM) used by others mostly in PC12 cells [20,23,56]. Considering tissue penetration and the purported levels of antipsychotics found in rodent brains [57], it is likely that upon repeated treatments, these drugs can reach levels that are sufficient to be neuroprotective.

## Conclusion

In conclusion, our data show that various D_2_-like receptor antagonists were able to protect primary hippocampal cultured neuronal cells against cell death induced by medium deprivation. Further studies are necessary to confirm the role of D_2_-like (D_2 _and/or D_4_) dopamine receptors and subsequent intracellular signaling pathways such as the inhibition of apoptosis-related effectors. Our findings also support the hypothesis that antipsychotics could modulate, via their neuroprotective properties, cognitive status in schizophrenic patients.

## Methods

### Materials

Materials used for cell cultures and Reverse Transcription-PCR were purchased from Invitrogen-Gibco BRL (Burlington, Ontario, Canada) and from Sigma Chemical Co. (Oakville, On, Canada). Haloperidol, (-)-eticlopride, raclopride, chlorpromazine and risperidone were obtained from Sigma Chemical Co. (Oakville, On, Canada). U 99194 maleate and L-741,742 hydrochloride were obtained from Tocris (Ellisville, MO, USA). The ginkgo biloba extract EGb 761 was kindly provided by IPSEN laboratories (Paris, France). Unless stated otherwise, other chemicals were purchased from Sigma-RBI (Natik, MA, USA). All drugs were freshly prepared on the day of the experiment in a final concentration of ethanol or DMSO that does not exceed 0.01%.

### Neuronal hippocampal cell cultures

Enriched neuronal hippocampal cells were prepared from E19 fetuses obtained from Sprague-Dawley rats (Charles River Canada, St-Constant, Québec, Canada) as described previously [58]. Animal care was according to protocols and guidelines of the McGill University Animal Care Committee and the Canadian Council for Animal Care.

Hippocampal cells were plated at day 0 at a density of approximately 12 × 10^4 ^viable cells per well in 96-well plates. They were grown in Dulbecco's modified Eagles medium (D-MEM) medium supplemented with 20 mM KCl, 15 mM HEPES and 1% (v/v) serum-free growth medium N2 (final composition: 5 μ g/ml insulin, 100 μM putrescine, 20 nM progesterone, 100 μg/ml transferrin, 30 nM selenium), and maintained at 37°C in a 95% air/5% CO_2 _humidified atmosphere during 3 days.

### Immunochemistry

On day 0, hippocampal neurons were plated on poly d-lysine (25 μg/mL)-coated 12 mm glass coverslips (Fisher, Nepean, On, Canada) placed in multiwell plates and grown in the same medium as described above. On day 3, the medium was removed, the cells rinsed with PBS and fixed with 4% paraformaldehyde at room temperature (RT) for 15 min. Cells were pre-treated with 0.1% Triton X-100 for 20 min followed by a blocking step with 5% normal donkey serum (NDS)/bovine serum albumine (BSA) 5%/0.1% Triton X-100 in PBS for 20 min at RT. The cells were then incubated overnight at 4°C with a mouse anti- NeuN monoclonal antibody (1:250; Chemicon, Temecula, CA, USA) in PBS supplemented with 0.1% Triton X-100, NDS (5%) and BSA (0.5%). After several washes in PBS, the secondary antibody (Alexa Fluor 568 goat anti-mouse IgG_1_, 1:200; Invitrogen) diluted in the same buffer as the primary antibody was added and incubation proceeded for 2 hrs at RT. The coverslips were washed several times then mounted on slides with DAPI-containing Vectashield (Vector Laboratories, Burlington, On, Canada). Hippocampal cells were examined using conventional immunofluorescence microscopy and counted from three 40× magnification fields on one slide for each experimental condition. Each experiment was repeated using a different culture preparation.

### Reverse Transcription-Polymerase Chain Reaction (RT-PCR)

RT-PCR was performed using a sensitive two-step PCR protocol according to [59] with some minor modifications. Total RNA was isolated from 3-day-old rat primary cultured hippocampal neurons (from two different experiments) and from rat striatum (P14) by using the Qiagen (Mississauga, On, Canada) RNeasy midi-kit in conjunction with the RNase-free DNase set according to the manufacturer's protocol. First strand cDNA was generated from 1 μg total RNA in a 20 μl reaction containing: 2.5 μM random hexamers (Applied Biosystems, Foster City, CA, USA), 10 mM DTT (Sigma), 20 U Ribonuclease Inhibitor (Takara Biomedicals, Otsu, Japan), 0.5 mM dNTP, 1X First strand buffer, and 100 U SuperScript II RNase H^- ^Reverse Transcriptase (all from Invitrogen). Following an overnight incubation at 42°C, the enzyme was denatured at 70°C and the RNA complementary to the cDNA was hydrolysed with 2U RNaseH (Takara Biomedicals) for 20 min at 37°C. Reactions in which the reverse transcriptase was omitted were run in parallel as controls for any residual genomic DNA.

In the first step PCR, cDNAs for dopamine receptor subtypes D_1 _to D_5 _were amplified simultaneously from 2 μl of each reverse transcription reactions in 20 cycle multiplex reactions (mCPR). This was followed by a second round of 35 cycles PCR in which individual cDNAs (D_1 _to D_5_) were amplified separately in reactions using 2% of the first round products as substrate. All PCR amplifications (94°C, 30 s; 60°C, 30 s; 72°C, 35 s) were performed in a 96-well thermocycler (GeneAmp 9700, Applied Biosystems). The final reaction volume for each amplification reaction was 100 μl and contained 1× PCR buffer, 2 mM MgCl_2_, 200 μM dNTP, 1 U Platinum Taq DNA polymerase (all from Invitrogen), and 10 pmoles of each selected forward and reverse primers. Primer pairs (custom-synthesized by Invitrogen) for D_2_-like dopamine receptor subtypes D_2_, D_3_, and D_4 _were designed to flank at least one intron according to the NCBI GenBank sequence database and to lie outside regions of significant homology. Likewise, primer pairs amplifying sequences from intronless coding regions of D_1_-like (D_1 _and D_5_) receptor subtypes were derived from regions of low homology. Primer positions for D_2 _or D_3 _were chosen in the vicinity of those used by [60] to detect possible alternative splicing isoforms.

The following oligonucleotide primers were used (the predicted size for PCR products are given in parentheses): receptor D_1_, forward 5'-CATCACCTTCGATGTGTTTGTGTG-3' and reverse 5'-GCTATTCCACCAGCCTCTTCCTT-3' (300 bp); receptor D_2_, forward 5'-GCCAACCCTGCCTTTGTGGT-3' and reverse 5'-GCTTTCTGCGGCTCATCGTCT-3' (538 bp and 451 bp); receptor D_3_, forward 5'-GCCTGGTATGTGCTGCTGTGCT-3' and reverse 5'-CGTTTTCTTTGCCTTTGCCTCA-3' (523 bp and 410 bp); receptor D_4_, forward 5'-TCTACTCCGAGGGTGGCGTGT-3' and reverse 5'-GCAGGAAGAAGGAACAAATGGATG-3' (324 bp); receptor D_5_, forward 5'-GGAGGAAGGCTGGGAGCTAGAA-3' and reverse 5'-GCTGACACAAGGGAAGCCAGTC-3' (403 bp).

Fifteen μl of each second round PCR were analyzed on a 2% agarose gel with 1 μg of molecular size standards (Invitrogen). Discrimination between potential amplification of genomic DNA sequences and RT-PCR on mRNA was based on the size of the PCR product (in the case of D_2_, D_3_, and D_4 _receptors) and on the absence of a PCR product when reverse transcriptase was omitted (for all 5 subtypes). PCR products of the anticipated sizes were then purified with the QIAquick PCR purification kit (Qiagen), and sequenced at Laval University's Service d'Analyse et de Synthèse SCF Facility (Québec, Canada) to ensure they matched the respective known cDNA sequences.

### Toxicity induced by growth medium deprivation

At day 3 of plating, the medium was removed and cells were incubated at 37°C in D-MEM medium supplemented with 15 mM HEPES and 5 μg/ml insulin and devoid of putrescine, progesterone, transferrin, selenium and KCl. Cells were then treated with either vehicle or different drugs. Neuronal viability was determined 3 days later using the MTT and neutral red (NR) colorimetric assays (see below).

### Assessment of neuronal survival

Neuronal survival was estimated using the MTT [3-(4,5-dimethylthiazol-2-yl)-2,5-diphenyl tetrazolium bromide] and NR [3-amino-7-dimethyl-amino-2-methylphenazine hydrochloride] dyes, which are respectively indicators of mitochondrial activity and lysosomal uptake of living cells. Cell survival was spectrophotometrically determined at 570 nm (for MTT assay) and 540 nm (for NR assay) using a micro-plate reader (Bio-Tek Instruments^® ^Inc., Ville St-Laurent, Québec, Canada) [58].

### Assessment of intracellular reactive oxygen species

Dichlorofluorescein (DCF) fluorescence assay was used to determine the intracellular production of reactive oxygen species [58]. Briefly, cells were treated with the cell permeable 2,7-dichlorofluorescein diacetate (DCFH-DA; Molecular Probes Inc., Eugene, OR) which is converted into 2',7'-dichlorofluorescein. 2',7'-dichlorofluorescein is then able to interact with intracellular peroxides to form the highly fluorescent compound DCF. The medium was removed 3 days after plating and replaced with fresh medium containing 15 mM HEPES, 5 μg/ml insulin and 5 μM DCFH-DA in the presence of absence of either haloperidol (1 μM) or EGb 761 (50 μg/ml). DCF fluorescence was quantified (excitation = 485 nm, emission = 530 nm) the day after using a fluorescence multiwell plate reader (Bio-Tek Instruments^® ^Inc., Ville St-Laurent, Québec, Canada).

### Statistical analyses

Optical density (OD) reflecting MTT reduction and NR intake into intact cells, was proportional to the number of viable cells. The OD of the control group (CT, i.e. the group of non-treated cells deprived during 3 days with growth medium) was regarded as 100%. The rate of surviving cells treated with various drugs during 3 days was expressed as percent of control groups. Statistical analysis was performed using one-way ANOVA followed by a Newman Keuls' multiple comparison test with p < 0.05 being considered statistically significant. An unpaired t-test was used to compare reactive oxygen species production (as estimated by the DCF assay) between control group and groups treated with drugs, survival of cells treated with clozapine alone and cells treated with raclopride and clozapine (Table 2), and survival of non-treated cells and cells treated with caspases (Table 3).

## Authors' contributions

SB carried out the cell cultures experiments and related experiments, performed the statistical analysis, and drafted the manuscript. MD carried out the immunochemistry and molecular biology (RT-PCR) studies and helped to draft the manuscript. FM carried out the preliminary RT-PCR studies. SW helped to draft the manuscript. RQ conceived the study, participated in its design and coordination and helped to draft the manuscript. All author(s) read and approved the final manuscript.

## Figures and Tables

**Table 1:** Comparison of the affinities (Ki values, nM) of various receptor antagonists at dopamine D_2 _and D_4 _receptors with their protective effects (at 10^-6^M) on hippocampal neurons (expressed in % of MTT values vs control group).

	D_2 _subtype Affinity (Ki, nM)	D_4 _subtype Affinity (Ki, nM)	Neuroprotective activity (% vs control, MTT values)
(+)-butaclamol	0.8	40	464
Clozapine	36–190	1.6–40	340
Chlorpromazine	0.66–1.5	1.15–37	320
Domperidone	0.30	ND	299
Haloperidol	0.35–1	0.84–5.1	271
(±)-sulpiride ^a^	9.2–15	52–1000	199
L-741,742	> 1700	3.5	186
Risperidone	0.3–5.9	0.25–16	162
Haloperidol metabolite	> 10000	ND	108
Raclopride	0.64–1.8	237–620	110
Remoxipride	30–2000	2800–3690	108
U 99194	2281	> 10000	100

Data from [30-32, 39-45]. ND, not determined.

^a ^S-(-)-sulpiride.

**Table 2:** Effects of NE-100, (+)-MK-801 and of a co-treatment of raclopride with clozapine in enriched hippocampal neuronal cultures, as estimated by the MTT and NR assays

Drugs	MTT (% of control)	NR (% of control)
Control	100 ± 6	ND
+ N2	177 ± 10	ND
+ Raclopride (10^-6 ^M)	103 ± 5	ND
+ Clozapine (10^-6 ^M)	199 ± 13	ND
+ Raclopride (10^-6 ^M) + Clozapine (10^-6 ^M)	117 ± 8^¶^	ND
Control	100 ± 6	100 ± 4
+ NE-100 (10^-7 ^M)	97 ± 6	93 ± 3
+ NE-100 (10^-6 ^M)	112 ± 9	95 ± 4
+ NE-100 (10^-5 ^M)	127 ± 9	82 ± 5*
Control	100 ± 5	100 ± 8
+ (+)-MK-801 (10^-6 ^M)	123 ± 9	128 ± 6
+ (+)-MK-801 (10^-5 ^M)	130 ± 12	112 ± 14

Data represent mean ± SEM of at least three separate experiments. *p < 0.01, control versus drugs-treated cells. ^¶^p < 0.01, cells treated with clozapine alone versus cells co-treated with raclopride and clozapine. ND: not determined.

**Table 3:** Effects of inhibitors of caspases 3, 8 and 9 against toxicity and ROS accumulation after serum deprivation in enriched hippocampal neuronal cultures, as estimated by MTT and DCF assays, respectively.

Drugs	MTT values (% of control)	DCF values (% of control)
Control	100 ± 4	100 ± 6
+ Inhibitor of caspase 3 (DEVDO-CHO, 5 μM)	171 ± 12 *	85 ± 4
+ Inhibitor of caspase 8 (IETD-CHO 5 μM)	141 ± 9 *	96 ± 5
+ Inhibitor of caspase 9 (LEHD-CHO, 5 μM)	135 ± 10 *	89 ± 6
+ Clozapine (1 μM)	140 ± 8 *	ND

Data represent mean ± SEM of at least three separate experiments. *p < 0.01 compared to vehicle-treated cells. ND: not determined
